# Supplementary material for: Treatment of Periprosthetic Joint Infection with Intravenous Vancomycin: Do We Hit the Target?
Source: Antibiotics (Basel). 2024 Dec 18;13(12):1226. doi: 10.3390/antibiotics13121226 (PMC11727632; doi:10.3390/antibiotics13121226)
Supplement: Supplementary file 1 [file antibiotics-13-01226-s001.zip › antibiotics-3339982-supplementary.pdf]

Additional tables display the isolated organisms from all cultures collected from the patients in the study, along with the number of patients in which they were detected.

Table S1. Organisms isolated from synovial fluid cultures

Anaerococcus spp	1
Enterococcus faecium	1
Escherichia coli (E.Coli)	1
Staphylococcus aureus	5
Staphylococcus capitis	1
Staphylococcus epidermidis	4
Staphylococcus lugdunensis	4
Staphylococcus simulans	1
Streptococcus agalactiae	1
Streptococcus dysagalactiae	2
Streptococcus mitis	2

Table S2. Organisms isolated from tissue cultures from the surgeries.

Alpha-hemolytic streptococci	1
Anaerococcus murdochii	1
Bacillus	1
Corynebacterium	6
Cutibacterium acnes/Propionibacterium acnes	7
Enterobacter cloacae	1
Enterococcus faecalis	7
Enterococcus faecium	1
Escherichia coli (E.Coli)	3
Finegoldia magna/Peptostreptococcus magnus	8
Klebsiella oxytoca	2
Klebsiella pnemoniae	1
Pseudomonas aeruginosa	1
Staphylococcus aureus	24
Staphylococcus capitis	16
Staphylococcus caprae	1
Staphylococcus epidermidis	30
Staphylococcus lugdunensis	8
Streptococcus agalactiae	1
Streptococcus dysagalactiae	5
Streptococcus sanguinis	1

Table S3. All organisms collected regardless location.

Alpha-hemolytic streptococci	1
Anaerococcus spp	1
Anaerococcus murdochii	1
Bacillus	1
Corynebacterium	6
Cutibacterium acnes/Propionibacterium acnes	7
Enterobacter cloacae	1
Enterococcus faecalis	7
Enterococcus faecium	1

Escherichia coli (E.Coli)	3
Finegoldia magna/Peptostreptococcus magnus	8
Klebsiella oxytoca	2
Klebsiella pnemoniae	1
Pseudomonas aeruginosa	1
Staphylococcus aureus	25
Staphylococcus capitis	16
Staphylococcus caprae	1
Staphylococcus epidermidis	30
Staphylococcus lugdunensis	10
Staphylococcus simulans	1
Streptococcus agalactiae	1
Streptococcus dysagalactiae	5
Streptococcus mitis	2
Streptococcus sanguinis	1
